# Granulomatous tattoo reaction associated with poly (lactic-co-glycolic acid) (PLGA) microsphere-based naltrexone injections

**DOI:** 10.1016/j.jdcr.2025.03.008

**Published:** 2025-03-24

**Authors:** Eva Shelton, Charlotte LaSenna, Bridget Shields, Connie Wang

**Affiliations:** Department of Dermatology, University of Wisconsin, Madison, Wisconsin

**Keywords:** granuloma, granulomatous tattoo reaction, microsphere, PLGA microsphere

## Introduction

Granulomatous tattoo reactions can occur due to chronic exposure to tattoo ink and often require evaluation to distinguish them from sarcoidosis, infection, or foreign body granulomas. Contributing factors include variations in tattoo pigments and differences in patient-specific immune responses. Medications modulating immune function, such as tumor necrosis factor inhibitors and immune checkpoint inhibitors, have also been implicated.^1^, ^2^, ^3^

Additionally, microsphere technology, which utilizes small spherical particles for drug delivery, has been associated with localized granulomatous reactions. Among these, poly (lactic-co-glycolic acid) (PLGA)-based microspheres have emerged as a prominent drug delivery platform due to their biodegradability, biocompatibility, and controlled release. This technology facilitates the delivery of diverse therapeutic agents, including small molecules, peptides, proteins, and DNA, while minimizing dosage requirements and adverse effects.^4^ However, localized granulomatous responses to PLGA microspheres have been documented, particularly with cosmetic fillers.^5^

While previously reported granulomatous reactions have been localized to injection sites, we report a case of widespread granulomatous tattoo reaction potentially associated with the extended-release naltrexone formulation (Vivitrol) utilizing PLGA microsphere technology. This case raises the possibility that microsphere drug delivery systems could contribute to immune-mediated reactions, emphasizing the need for additional research to better understand their mechanisms and potential effects.

## Case report

A 26-year-old woman with no history of granulomatous disorders who previously tolerated oral naltrexone developed nodules in her tattoos after initiating Vivitrol, a PLGA microsphere formulation of extended-release naltrexone that is injected intramuscularly every 4 weeks. The patient had several black ink tattoos of varying ages, ranging from 4 to 7 years old.

Three weeks after her first Vivitrol injection to the buttocks, an area free of tattoos, she noted small, pruritic papules confined to her most recent tattoo on her arm. Self-treatment with cold compresses and over-the-counter 1% hydrocortisone provided minimal relief. With each subsequent injection, she observed enlargement of existing papules, appearance of new papules in other tattoos, worsening pruritus, and induration of tattoo lines. The progression of her symptoms led to her presentation to a dermatology clinic.

**Fig 1:**
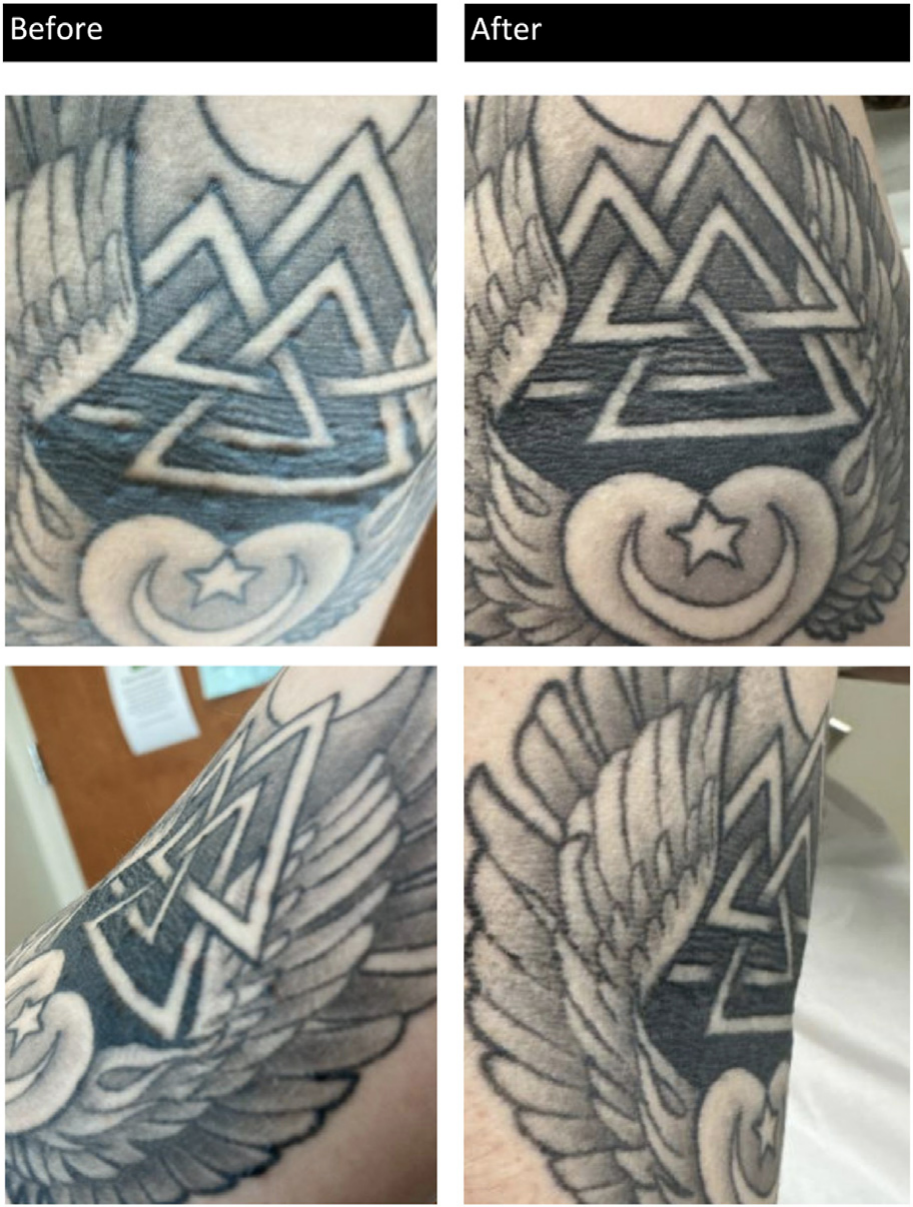
Before (initial presentation): Firm papules without epidermal change, confined within indurated tattoo lines. After (3 month follow-up): Flattening of the papules with less induration of the tattoo.

**Fig 2:**
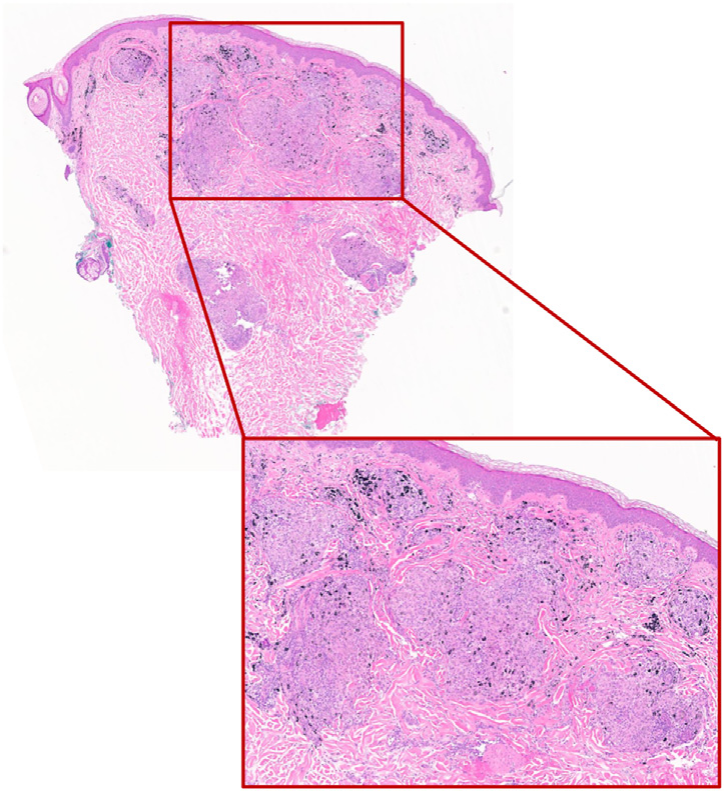
Biopsy revealed collections of epithelioid histiocytes forming granulomas in association with tattoo pigment and patchy lymphocytic inflammation, consistent with a granulomatous tattoo reaction.

Examination revealed firm papules and nodules within indurated tattoo lines without epidermal change (Fig 1). A biopsy demonstrated collections of epithelioid histiocytes forming granulomas in association with tattoo pigment and patchy lymphocytic infiltrate, consistent with a granulomatous tattoo reaction (Fig 2). Infectious workup, including acid-fast bacilli, Grocott methenamine silver, periodic acid–Schiff with diastase, and gram stains, was negative. Additionally, workup for sarcoidosis, including chest X-ray and serum angiotensin-converting enzyme, was unremarkable. The patient was treated with high-potency topical corticosteroids and discontinued Vivitrol while remaining off oral naltrexone. She did not resume oral naltrexone following the discontinuation of Vivitrol. At her 1-month follow-up, she reported significant symptomatic improvement, with flattening of papules and reduced induration within tattoo lines. At her 3-month follow-up, her granulomatous tattoo reaction had fully resolved (Fig 1).

## Discussion

This case represents a widespread granulomatous tattoo reaction in association with PLGA microsphere technology. Although a true causal relationship cannot be directly inferred, the temporal relationship between Vivitrol administration and symptom onset, combined with rapid resolution following discontinuation, suggests PLGA microspheres played a role in triggering the reaction. Symptoms worsening with each injection and improving upon cessation support this association. Additionally, the patient’s prior tolerance of oral naltrexone suggests that naltrexone was not the inciting factor. However, it remains unknown whether reintroducing oral naltrexone after the onset of granulomatous inflammation would have provoked a recurrent reaction. Further investigation into the potential for cross-reactivity between different formulations of naltrexone in granulomatous hypersensitivity is warranted.

PLGA microspheres are increasingly used for controlled and sustained drug delivery, although their potential to provoke granulomatous inflammation in susceptible individuals warrants attention. While localized granulomatous reactions to microsphere drug delivery systems, such as cosmetic fillers using polymethylmethacrylate microspheres and other medications utilizing PLGA microspheres including leuprolide,^6^ have been documented, these reactions are typically confined to injection sites. It has been proposed that these microspheres may alter the local immune response in the skin, predisposing patients to granulomatous foreign body reactions.^7^

In this case, however, the granulomatous reaction was unique in that it affected tattooed skin at multiple sites far from the injection site. The systemic nature of the reaction suggests a broader immune-mediated process rather than direct migration of the microspheres. There have been few reports of tattoo granulomas developing in the setting of systemic immune-modulating medications, such as tumor necrosis factor-α inhibitors, immune checkpoint inhibitors, and even COVID vaccines. These agents may modulate immune responses, leading to granuloma formation, though the precise mechanisms remain poorly understood and require further research.^1^, ^2^, ^3^

Granulomatous tattoo reactions often mimic cutaneous sarcoidosis. In rare cases, they may even precede systemic sarcoidosis, although systemic involvement is uncommon.^8^ Some suggest granulomatous tattoo reactions represent localized sarcoidal reactions, while others consider them distinct entities.^9^^,^^10^ The overlap in clinical and histologic features highlights the importance of thorough evaluation to rule out systemic disease.

This case highlights granulomatous tattoo reactions as a potential adverse effect of long-acting injectable medications utilizing microsphere technology. It also emphasizes the importance of distinguishing these reactions from other causes of granulomatous inflammation, such as infection or sarcoidosis, through detailed clinical and histologic evaluation.

In conclusion, this case contributes to the growing body of literature by identifying PLGA microspheres as a potential systemic trigger for granulomatous tattoo reactions. Further research is needed to clarify the prevalence of this condition, the mechanisms underlying these immune-mediated responses, and the factors that predispose certain patients to such reactions. Clinicians should maintain an index of suspicion for medication-induced granulomatous reactions, particularly when new-onset tattoo changes occur in temporal association with systemic therapies.

## Conflicts of interest

None disclosed.

## References

[1] Bachmeyer C., Blum L., Petitjean B., Kemiche F., Pertuiset E. (2007). Granulomatous tattoo reaction in a patient treated with etanercept. J Eur Acad Dermatol Venereol.

[2] Kluger N. (2019). Tattoo reactions associated with targeted therapies and immune checkpoint inhibitors for advanced cancers: a brief review. Dermatology.

[3] Haller C.N., Schnebelen A., Cadmus S.D. (2023). Sarcoidal tattoo granuloma after COVID-19 vaccine. JAAD Case Rep.

[4] Su Y., Zhang B., Sun R. (2021). PLGA-based biodegradable microspheres in drug delivery: recent advances in research and application. Drug Deliv.

[5] Patel B.P. (2020). PMMA safety for facial filling: review of rates of granuloma occurrence and treatment methods. Aesthet Plast Surg.

[6] Yasukawa K., Sawamura D., Sugawara H., Kato N. (2005). Leuprorelin acetate granulomas: case reports and review of the literature. Br J Dermatol.

[7] Huynh T.N., Jackson J.D., Brodell R.T. (2014). Tattoo and vaccination sites: possible nest for opportunistic infections, tumors, and dysimmune reactions. Clin Dermatol.

[8] Bose R., Sibley C., Fahim S. (2020). Granulomatous and systemic inflammatory reactions from tattoo ink: case report and concise review. SAGE Open Med Case Rep.

[9] Antonovich D.D., Callen J.P. (2005). Development of sarcoidosis in cosmetic tattoos. Arch Dermatol.

[10] van der Bent S.A.S., Rauwerdink D., Oyen E.M.M., Maijer K.I., Rustemeyer T., Wolkerstorfer A. (2021). Complications of tattoos and permanent makeup: overview and analysis of 308 cases. J Cosmet Dermatol.

